# Outbreak of Septic Arthritis Associated with Intra-Articular Injections at an Outpatient Practice — New Jersey, 2017

**DOI:** 10.15585/mmwr.mm6629a3

**Published:** 2017-07-28

**Authors:** Kathleen Ross, Jason Mehr, Barbara Carothers, Rebecca Greeley, Isaac Benowitz, Lisa McHugh, David Henry, Lisa DiFedele, Eric Adler, Shereen Naqvi, Edward Lifshitz, Christina Tan, Barbara Montana

**Affiliations:** ^1^New Jersey Department of Health; ^2^Applied Epidemiology Fellowship Program, Council of State and Territorial Epidemiologists; ^3^Division of Healthcare Quality Promotion, National Center for Emerging and Zoonotic Infectious Diseases, CDC; ^4^Monmouth County Regional Health Commission No. 1.

On March 6, 2017, the New Jersey Department of Health (NJDOH) was notified of three cases of septic arthritis in patients who had received intra-articular injections for osteoarthritic knee pain at a private outpatient practice. The practice voluntarily closed the next day. NJDOH, in conjunction with the local health department and the New Jersey Board of Medical Examiners, conducted an investigation and identified 41 cases of septic arthritis associated with intra-articular injections administered during 250 patient visits at the same practice, including 30 (73%) patients who required surgery. Bacterial cultures of synovial fluid or tissue from 15 (37%) patients were positive; all recovered organisms were oral flora. An infection prevention assessment of the practice identified multiple breaches of recommended infection prevention practices, including inadequate hand hygiene, inappropriate use of pharmacy bulk packaged (PBP) products as multiple-dose containers and handling PBP products outside of required pharmacy conditions, and preparation of syringes up to 4 days in advance of their intended use. No additional septic arthritis cases were identified after infection prevention recommendations were implemented within the practice.

## Investigation and Response

On March 6, 2017, Monmouth County Regional Health Commission No. 1 (MCRHC) notified NJDOH that three patients were hospitalized for septic arthritis after receiving intra-articular injections for osteoarthritis pain relief at practice A, a private outpatient facility where procedures were performed by two staff physicians with the aid of two medical assistants. On March 7, practice A voluntarily closed in response to a large number of reports of severe knee pain and swelling. On March 8, NJDOH notified the New Jersey Board of Medical Examiners, which oversees physician licensure, to facilitate and coordinate a joint investigation.

A confirmed case of septic arthritis was defined as any one of the following in a patient who received intra-articular injections at practice A during March 1–6, 2017: 1) isolation of any microorganism from synovial fluid or tissue collected from the injected joint, 2) positive Gram stain of synovial fluid, 3) synovial fluid white blood cell count of >20,000/mm^3^, and 4) recipient of intravenous antibiotics or surgical debridement for a clinical diagnosis of septic arthritis.

Among 250 patient visits involving knee intra-articular injections at practice A during March 1–6, NJDOH identified 41 confirmed cases (16%) of septic arthritis. Patients had been scheduled over 3 consecutive clinic days (March 1, March 2, and March 6) with no apparent clustering by appointment time; the same physician administered all injections on these 3 days. Information on time of symptom onset was available for 38 (93%) of 41 patients and ranged from zero to 65* days after injection; 35 (92%) of the 38 patients developed symptoms within 48 hours of the procedure. Thirty (73%) of the 41 patients required surgery.

All 41 patients had synovial fluid or knee tissue obtained during surgery collected for culture, and cultures were positive for 15 (37%) patients. Bacteria recovered included *Streptococcus mitis-oralis* (10 patients), *Abiotrophia defectiva* (two), *Staphylococcus aureus* (two), *Actinomyces odontolyticus* (one), alpha-hemolytic *Streptococcus* (one), *Eikenella corrodens* (one), *Haemophilus parainfluenzae* (one), *Neisseria oralis* (one), *Streptococcus gordonii* (one), *Streptococcus intermedius-milleri* (one), *Streptococcus sanguinis* (one), and *Veillonella* (one); five patients had polymicrobial infections. Cultures from 26 (63%) patients were negative. All recovered organisms are commonly found in oral flora (*1*,*2*). In addition to bacteria recovered from culture of synovial fluid or tissue, *Staphylococcus aureus* was isolated from the blood of two patients.

On March 13, MCRHC, NJDOH, and the New Jersey Division of Consumer Affairs representing the New Jersey Board of Medical Examiners conducted an unannounced visit to practice A to inspect the premises, interview staff members, observe infection prevention practices, and review records. Because the practice remained closed to patients at this time, mock procedures were observed during the visit.

Multiple breaches in infection prevention recommendations were identified. Staff members did not have access to a handwashing sink, and alcohol-based hand rub was not available in medication preparation or treatment areas. Staff members, operating under the mistaken belief that PBP products could be used as multiple-dose containers outside of pharmacy conditions (e.g., use of a laminar flow hood, appropriate garbing, staff training, and environmental monitoring), accessed a 50 mL PBP container of contrast material up to 50 times to prepare syringes for multiple patients, with the septum of the container cleaned with alcohol only before the initial draw. Staff members prepared injections in a separate room, away from the patient treatment area; however, pharmacy conditions necessary for batch preparation of syringes and use of PBP products were not in place. In addition, injectable medications were drawn into syringes by medical assistants up to 4 days in advance of procedures, contrary to the recommended practice of administering medication from single-dose vials within 1 hour of preparation (*3*).

Injections were initiated using a needle and syringe filled with local anesthetic. After injecting the anesthetic, the physician removed the syringe, leaving the needle within the intra-articular space. A second syringe containing contrast material from the PBP container was then attached to the needle hub and used to facilitate fluoroscopic needle placement. This was followed by replacement with a third syringe containing a glucocorticoid or hyaluronic acid–based product. The physician did not wear a face mask during joint injection procedures and used nonsterile gloves to manipulate the needle hub during procedures.

Practice A was advised to immediately stop batch preparation of syringes and use of PBP products for multiple patients and to hire an infection preventionist to assess staff competency and ensure that hand hygiene, standard precautions, and safe injection practices were followed. No additional cases occurred after these measures were implemented.

## Discussion

An investigation of 41 cases of septic arthritis associated with intra-articular injections at an outpatient practice in New Jersey identified multiple breaches of recommended infection prevention practices during the preparation and administration of PBP products, which are intended for use in a pharmacy setting, using standards outlined by the United States Pharmacopeial Convention (USP) (*3*,*4*). PBP products are restricted to preparation of admixtures only in a suitable work area as defined by USP, such as in a laminar flow hood, and handled in accordance with sterile compounding standards outlined by the manufacturer and USP (*3*,*4*). CDC guidelines call for medications labeled as “single-dose” or “single-use” to be used for only one patient (*5*,*6*). Single-use medications, including PBP products, typically lack antimicrobial preservatives and can become contaminated and serve as a source of microorganisms when handled inappropriately (*6*). Use of a PBP product as a multiple-dose container outside of pharmacy conditions could contaminate the container and serve as a source of pathogens for multiple patients. Because practice A used a single PBP container of contrast material for as many as 50 patients, contamination of only a single container could account for the large number of cases identified in this outbreak.

Proper hand hygiene should be performed before handling any medications. In addition, batch preparation of medication for future administration should be performed in accordance with sterile compounding standards recommended by USP (*3*).

In this outbreak, all pathogens isolated were oral flora. CDC recommends that health care personnel wear face masks for spinal injection procedures that require injection of material or insertion of a catheter into epidural or subdural spaces (e.g., myelogram, administration of spinal or epidural anesthesia, or intrathecal chemotherapy) (*5*). Multiple outbreaks have demonstrated the risk for bacterial meningitis associated with droplet transmission of oral flora from health care personnel to patients during spinal injection procedures. The Association for Professionals in Infection Control and Epidemiology recommends the use of a face mask to contain respiratory droplets when preparing and injecting material into an intra-articular space (*7*). The use of multiple syringes with a single intra-articular needle could serve as a conduit for organisms to enter directly into the joint space if the needle hub is left exposed to potential respiratory droplets. Although this is a potential mechanism for a single case, it is unlikely to explain the large number of cases identified in this outbreak.

No additional septic arthritis cases were identified after infection prevention recommendations were implemented within the practice. The findings from this investigation highlight the need for better adherence to and oversight of basic infection prevention recommendations and sterile compounding standards in outpatient settings (*8*,*9*).

**Summary**

**What is already known about this topic?**

Single-use medications, including pharmacy bulk packaged (PBP) products, typically lack antimicrobial preservatives and can become contaminated and serve as a source of microorganisms when handled inappropriately. Use of a PBP product as a multiple-dose container outside of pharmacy conditions could contaminate the container and serve as a source of pathogens for multiple patients.

**What is added by this report?**

In March 2017, an outbreak of 41 cases of septic arthritis associated with intra-articular injections administered at an outpatient practice occurred in New Jersey. A public health investigation identified multiple breaches of recommended infection prevention practices during the preparation and administration of PBP products, which are intended for single-use, in accordance with standards outlined by the United States Pharmacopeial Convention.

**What are the implications for public health practice?**

No additional septic arthritis cases were identified after infection prevention recommendations were implemented within the practice. The findings from this investigation highlight the need for better adherence to and oversight of basic infection prevention recommendations and sterile compounding standards in outpatient settings.

## Footnotes

* One patient received an injection on March 1 but did not develop symptoms and seek medical care until the first week of May. This patient was reported to investigators by the hospital infection preventionist.
