# *Erratum*: Vol. 66, No. 29

**DOI:** 10.15585/mmwr.mm6714a5

**Published:** 2018-04-13

In the report “Outbreak of Septic Arthritis Associated with Intra-Articular Injections at an Outpatient Practice — New Jersey, 2017,” the type of container was misstated. The container should have been described as **a single-dose container** instead of a pharmacy bulk packaged (PBP) product. Single-dose containers can only be repackaged for multiple patients by qualified health care personnel in accordance with standards in United States Pharmacopeia General Chapter <797> Pharmaceutical Compounding — Sterile Preparations.

